# Remote Water Temperature Measurements Based on Brillouin Scattering with a Frequency Doubled Pulsed Yb:doped Fiber Amplifier

**DOI:** 10.3390/s8095820

**Published:** 2008-09-22

**Authors:** Kai Schorstein, Alexandru Popescu, Marco Göbel, Thomas Walther

**Affiliations:** TU Darmstadt, Institute for Applied Physics, Schlossgartenstr. 7, D-64289 Darmstadt, Germany; http://www.lqo.tu-darmstadt.de

**Keywords:** LIDAR, temperature profile, ocean, Brillouin scattering, remote sensing

## Abstract

Temperature profiles of the ocean are of interest for weather forecasts, climate studies and oceanography in general. Currently, mostly *in situ* techniques such as fixed buoys or bathythermographs deliver oceanic temperature profiles. A LIDAR method based on Brillouin scattering is an attractive alternative for remote sensing of such water temperature profiles. It makes it possible to deliver cost-effective on-line data covering an extended region of the ocean. The temperature measurement is based on spontaneous Brillouin scattering in water. In this contribution, we present the first water temperature measurements using a Yb:doped pulsed fiber amplifier. The fiber amplifier is a custom designed device which can be operated in a vibrational environment while emitting narrow bandwidth laser pulses. The device shows promising performance and demonstrates the feasibility of this approach. Furthermore, the current status of the receiver is briefly discussed; it is based on an excited state Faraday anomalous dispersion optical filter.

## 1. Introduction

**Figure 1.**
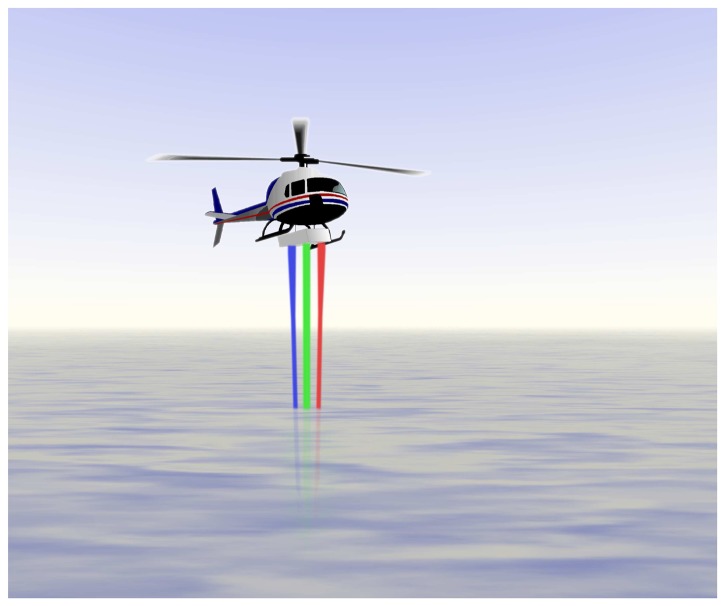
Vision of the Brillouin lidar mounted under a helicopter. The green ray depicts the laser pulse being sent into the ocean. The red and blue rays represent the two Brillouin shifted parts of the backscattered light whose frequencies are slightly red- and blue shifted with respect to the laser frequency.

The knowledge of temperature profiles of the upper-ocean mixed layer is highly relevant to oceanography. Furthermore, this data can provide valuable input for climate studies and weather forecasts. Currently, the measurement of temperature profiles is based on *in-situ* techniques such as fixed buoys or bathythermographs, which are deployed from aircrafts or ships. However, these techniques cannot deliver cost-effective on-line data from an extended region of the ocean. The exploitation of spontaneous Brillouin scattering for measuring sound speed and temperature in water was proposed by Hirschberg *et al.* [1] and later demonstrated by Guagliardo *et al.* using pulsed lasers [2] and Hirschberg *et al.* using cw-lasers [3]. In combination with remote sensing lidar techniques, a powerful instrument can be obtained that provides a much more efficient measurement technique compared to the established *in situ* techniques. In general, the lidar working principle is straight forward and is illustrated in Figure 1. A laser pulse is sent into the water. Brillouin scattering occurs in the water and the Brillouin backscattered light is analyzed by a detector unit. Depth resolution is achieved by analyzing the light for the return time. The temperature information is extracted from the spectral content of the signal. Thus, by careful analysis of the backscattered light for time-of-flight and frequency, the temperature profile in the ocean can be measured.

Specifically, light propagating in water is scattered off moving density fluctuations; due to the Doppler effect the spectrum of the backscattered light shows components shifted to the red and blue of the original light frequency. Thus, the method is sensitive to the local velocity of sound. The shifted frequency components are referred to as Brillouin lines. Frequency shifts for 532 nm-light for typical water temperatures and salinities found in the oceans are in the order of 7-8 GHz [4]. As the temperature dependence of the shift is known, the temperature information can be extracted from the data. The relation between the Brillouin shift ν_B_ and the velocity of sound v_S_ for backscattered light is given by [2]

(1) $$\nu_{B}=\pm 2\frac{n(S,T,\lambda )\;\upsilon_{s}(S,T)}{\lambda }.$$

The dependence of the refractive index n(S,T,λ) and of the sound velocity v_S_(S,T) on the salinity S, the temperature T and the wavelength λ are well known [4]. Relying on historical data for the salinity S and measuring the Brillouin shift ν_B_ enables us to deduce the temperature T. It should be noted that there is also a dependence on pressure, which has been omitted for clarity in the above equation, but which could be easily taken into account [4].

Laboratory based measurements by Fry and coworkers have shown the feasibility of this approach [5-7]. Theoretical investigations of the accuracy limitations have been performed [4]. It was found that an uncertainty of 1 MHz in the frequency shift measurement corresponds to an uncertainty in the temperature of 0.06 °C when the salinity is known. Assuming a world-wide constant salinity, averaged over historical data, a temperature accuracy of 0.5 °C is possible with a frequency uncertainty of 4 MHz. The maximum depth is limited by the available laser power, the detector sensitivity, the signal-to-noise ratio and the absorption in water. Estimates result in a penetration depth of up to 100 m [8]. The spatial resolution in the water is about a meter for pulse durations of 10 ns.

The necessary minimum specifications of the complete sensor system are rather stringent: (1) Since operation from a mobile platform is intended, the entire sensor has to be compact, insensitive to vibrations and exhibit low power consumption. (2) In order to provide 1 meter depth resolution, the laser pulse must have a temporal width less than about 9 ns. (3) In order to resolve the Brillouin shift the laser source must produce relatively high energy, narrow linewidth pulses close to the Fourier transform limit. (4) The laser radiation should be close to the absorption minimum of water, i.e. between 380 and 550 nm [9]. (5) The receiver unit must exhibit a high light gathering power, and be able to resolve the Brillouin-shift.

As proposed in earlier publications [10-15], a light source which is compatible with the above specification is a pulsed fiber amplifier. The fiber amplifier is composed of a master oscillator (seed) which defines spectral and temporal properties of the light. Then in a power amplifier the light is further amplified. Since there is no need for any resonant optics, the system is intrinsically insensitive to vibrations.

Different concepts for sensitive detection of the frequency shift are possible. The most straight forward solution is the use of an edge filter based on a Fabry-Perot interferometer [16], which is perfectly suitable for a laboratory. Carefully built, it possesses the required frequency resolution and the position of the Brillouin shifted lines can be accurately measured, but a Fabry-Perot and interferometers in general do not have the large acceptance angle required for practical use in the field and are also very sensitive to vibrations. Better alternatives are heterodyne detection [17] or edge filter techniques based on molecular or atomic absorption lines. The latter are static devices insensitive to vibrations capable of transforming a frequency shift into a change in transmission. Excited State Anomalous Dispersion Optical Filters (ESFADOF) are such static devices fulfilling the above requirements.

All the Brillouin-spectra measurements which are presented in the following chapter are performed with a scanning Fabry-Perot interferometer. An additional chapter is dedicated to the ESFADOF concept which documents the current status of development. The ESFADOF will be integrated into the whole system in the near future replacing the FPI detector.

## 2. The light source

Recent progress in fiber laser and amplifier technology has facilitated the extension to other operational wavelengths apart from the telecom wavelength region. High power systems have been realized by the introduction of large mode area fibers [18]. Fiber lasers and amplifiers have already proven their great versatility for delivering cw or pulsed radiation. Pulse lengths from femto- to nanoseconds have been realized [19-21]. In our application we are using a three stage Yb:doped fiber amplifier, which can be operated between 1020 nm-1100 nm when pumped at 976 nm [22]. This range makes it easier to match the laser with a receiver based on a molecular or atomic edge filter. Compared to a Nd:YAG laser, a fiber amplifier has significant advantages with regard to sensitivity to vibration, efficiency, weight, physical dimensions and operating wavelength region. The lower output energy obtained from the fiber amplifier could be balanced by the increased repetition rate of up to 5 kHz. Using a seeding technique near Fourier transform limited pulses can be generated [23]. These are required to accurately measure the peak positions of the Brillouin lines.

Energy scaling in this operating regime is in general limited by nonlinear effects. The high peak intensities inside the fiber core, the rather long interaction lengths and the small bandwidth promote the occurrence of stimulated Brillouin scattering (SBS) inside the fiber [24]. The onset of SBS can be prevented or at least shifted to higher energies by: (1) increasing the diameter of the fiber core thus reducing peak intensity, (2) reducing fiber length, (3) decreasing pulse length and (4) increasing bandwidth. Clearly, (3) and (4) are not options for our application. The fiber amplifier which is presented in this contribution has been optimized by using a highly doped and thus relatively short fiber as the third amplifier stage.

### Experimental Setup

**Figure 2.**
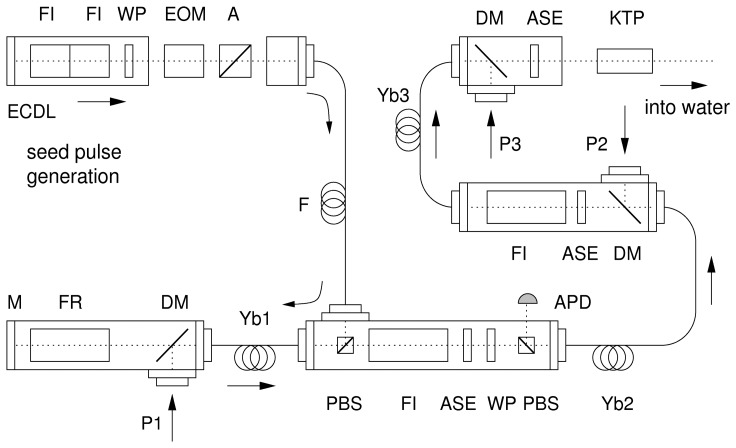
Setup of the fiber amplifier: Yb-doped fibers (Yb1-3), seed laser (ECDL), patch fiber (F), pump lasers (P1-3), electro-optic modulator (EOM), analyzer (A), polarizing beam splitter (PBS), mirror (M), Faraday isolator (FI), Faraday rotator (FR), λ/2-wave plate (WP), dichroic mirror (DM), interference filters (ASE), avalanche photo diode (APD) and nonlinear KTP crystal (KTP).

The experimental setup is shown in Figure 2 and consists of three main parts: the pulse generation unit, the amplifier itself and a second harmonic generation unit. The pulse generation unit consists of a small bandwidth external cavity diode laser (ECDL) that emits cw radiation at the desired wavelength. The diode is protected against back-reflections from the fiber amplifier by two Faraday isolators (FI). An electro-optic modulator (EOM) chops the cw-beam into laser pulses. At this point, the pulse duration and repetition rate are defined. Pulse durations between 10 ns and 1 μs at repetition rates of up to 5 kHz can be realized with our setup. The fiber amplifier can also be operated continuous wave (cw) depending on the position of the analyzer (A) behind the EOM. For adjustment, characterization and optimization of the receiver cw operation is advantageous.

The fiber amplifier is an advanced version of the amplifier described in ref. [8, 12, 21] and consists of three consecutive stages. Each stage uses its specific fiber with different geometry, length and dopant concentration. The properties of each fiber are listed in Table 1. Furthermore, the fiber ends are connectorized with high power, air-spaced SMA connectors and polished at an 8° angle to suppress spurious lasing. The first stage consists of a single-mode single-clad fiber while the second and third stage use a double-clad fiber with a multi-mode core.

The amplifier works as follows: A seed pulse arrives at the fiber amplifier and is reflected by the polarizing beam splitter (PBS) into the first stage (Yb1). After a first pass, the amplified pulse is reflected by a Faraday mirror (FR, M) and passes the fiber a second time. The Faraday isolator (FI) protects the first stage from unwanted back-propagating light. The second stage is passed only once. Again, a Faraday isolator (FI) separates the second and the third stage. After a final single-pass amplification in the third stage the high energy laser pulses will be frequency doubled in a KTP crystal (KTP). Interference band pass filters (ASE) are located at certain places to cut out unwanted amplified spontaneous emission.

### Results

The pulsed fiber amplifier was operated at 1064 nm with a pulse length of 10 ns. Different parameters affect the overall performance of the fiber amplifier: (1) The polarization of each stage has to be carefully controlled to reduce the loss at the Faraday isolators between the stages and to enhance the efficiency of the second harmonic generation after the third stage. (2) The beam profile has to be carefully adjusted since it is crucial for second harmonic generation efficiency. Both parameters can be controlled by carefully adjusting the coupling into the fiber. In general, it is favorably to excite only a few modes of the fiber. Each mode carries its own polarization and may contribute to a disturbed beam profile with inhomogeneous polarization.

The following characterization was done at a repetition rate of 1 kHz. The seed pulse energy which is launched into the first stage is estimated from the cw peak power and the temporal pulse shape to be 264 pJ. After the first stage a pulse energy of 0.86 μJ is measured, corresponding to a gain of 35.1 dB. The cw pump power of the first stage was approximately 300 mW. No signs of stimulated Brillouin scattering (SBS) or amplified spontaneous emission (ASE) are detectable. After the second stage up to 35.2 μJ are obtained, corresponding to a gain of 16.1 dB with a pump power of 6.4 W. This energy marks the SBS threshold. For stability reasons the second stage was operated below that threshold. Pulse energies of 34.6 μJ at 532 nm are obtained for effective pump powers of 5.7 W and 21.6 W at the second and third stage respectively.

For the actual measurements of the Brillouin spectra which are presented in the following chapter the fiber amplifier was operated well below the SBS threshold in order to improve stability and to avoid the risk of damaging the fibers. The Brillouin-spectrum measurements were performed at a repetition rate of 4.5 kHz with 17.7 μJ at 532 nm, which could be sustained for over 9 hours.

The bandwidths of the laser pulses were measured with a scanning Fabry-Perot to be 79.9 MHz; this is within a factor of two of the Fourier transform limit and is similar to prior experiments [23].

## 3. Brillouin scattering test facility

**Figure 3.**
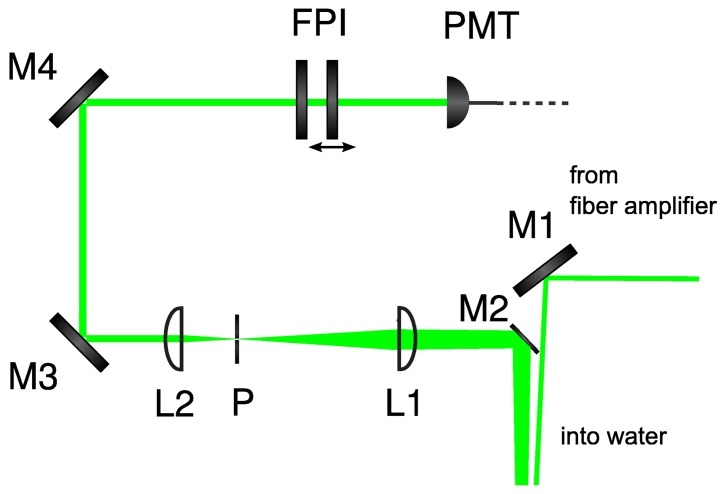
The transmission and receiving optics are composed of mirrors (M), lenses (L), pinhole (P), scanning Fabry-Perot interferometer (FPI) and a photomultiplier tube (PMT).

A water filled tube was used to evaluate the sensor system and to perform water temperature measurements. It consists of two segments divided by glass panes. These are inclined at a small angle in order to suppress back-reflections that disturb the measurements. Each segment can be stabilized to a different water temperature. The water tank is operated with double distilled water for which the salinity is virtually zero. Measurements with increased salinity are straightforward, since all mechanical parts are salt water resistant. The setup consists of the water tube as well as the transmission and receiving optics (cf. Figure 3). The light produced by the fiber amplifier is injected into the water by the mirror M1. The backscattered light is collected at a small angle to the backscattering direction (180°) by the mirror M2 and is directed through the receiving telescope. A pinhole (P) which is placed in the back focal plane of the first lens selects the effective collection volume inside the water tube. Then, the light is directed to the scanning Fabry-Perot interferometer (FPI). Finally, a photomultiplier (PMT) detects the light emerging from the FPI. Clearly, the current configuration is not able to measure range resolved Brillouin spectra, but spectra for a fixed distance can be obtained. The raw spectra taken from single FPI scans can be processed in order to extract the frequency shift information.

Since the free spectral range (FSR) of the FPI must be known in order to accurately determine the Brillouin shift, the following calibration procedure has been implemented. After all measurements have been performed with the pulsed fiber amplifier, the fiber amplifier is switched to the cw operation mode by rotating the analyzer behind the EOM. By rotating the mirror M2 at the receiving optics the cw light is directly sent through the telescope into the FPI. Then, the external cavity diode laser (ECDL) is scanned and resonance peaks can be observed with a separation of the FSR. Simultaneously resonance peaks are recorded from another Fabry-Perot interferometer with a known FSR of 1 GHz. By comparison of these two sets of frequency markers the FSR can be calibrated to yield 20.622(1) GHz. This is done by exploiting the symmetry of the Brillouin shift with respect to the Rayleigh peak; a quadratic function is found that can be used to correct the nonlinearity of the scan.

**Figure 4.**
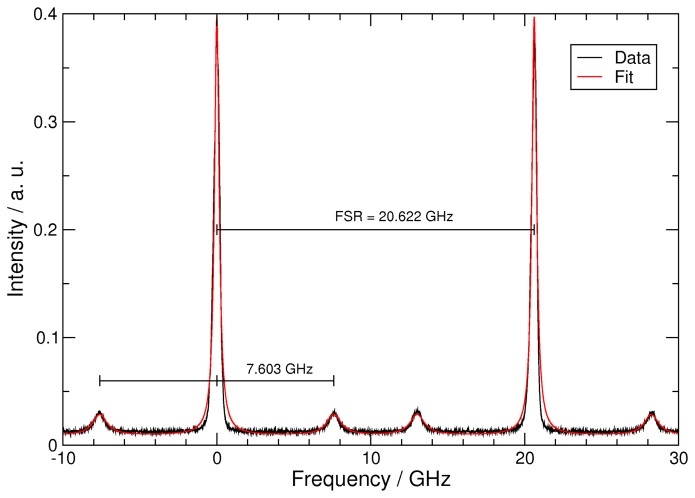
Measured Brillouin spectrum for a water temperature of 33.6 ±0.2 °C. The frequency shift resulting from the fitting procedure is 7.603 ±0.015 GHz.

The measurement of a single spectrum takes 50 seconds. Figure 4 shows a measured and calibrated spectrum recorded for a water temperature of 33.6 ±0.2 °C; it is composed of about 140,000 laser pulses. The Airy peak corresponding to the Rayleigh backscattered component appears twice, once in each order of the Fabry-Perot as it is scanned through two successive orders. The four smaller peaks originate from the Brillouin backscattered light; they are symmetrically located on either side of their respective Rayleigh peaks. A moving average of 10 was applied to smooth the data. A Levenberg-Marquardt algorithm was implemented to provide a nonlinear least-square fit to these data using an Airy function for the Rayleigh peaks and Lorentz functions for the Brillouin lines. The Brillouin frequency shifts can be obtained from these fits. The fitted function in Figure 4 is indicated by the red curve. The Rayleigh peaks are relatively large, since we have made no effort to remove hydrosols from the distilled water; in fact, we have not changed the water during the past year.

**Figure 5.**
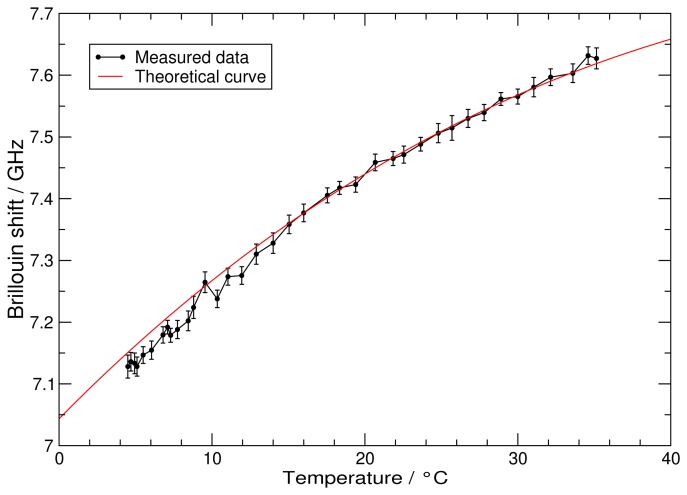
Measurement of the frequency shift versus the water temperature. The red curve indicates the theoretical frequency shift at zero salinity after Fry *et al.* [2].

The results for a series of different water temperatures are compiled in Figure 5. Except for some outliers the agreement between the measured and theoretically calculated [4] frequency shifts is excellent. The outliers are explained by systematic problems with single scans, which could be due to energy fluctuations of the fiber amplifier. The systematic deviation of the data below 15 °C is due to problems achieving a homogeneous temperature distribution in the water tank.

Uncertainties in the measurement of the position of the Brillouin peaks directly affect the temperature resolution. When the salinity is zero, a position uncertainty of 1 MHz corresponds to a temperature resolution of 0.04 °C [4].

Converting the mean error of all measurements, 14.4 MHz, into an uncertainty in temperature results in an accuracy of 0.57 °C for the current setup. The following improvements are about to be implemented in the near future in order to increase the temperature accuracy: (1) The energy of each pulse will be monitored and used to correct the data. (2) The stability of the FPI will be improved to increase the frequency resolution. (3) The temperature stabilization of the water tank will be improved to minimize systematics.

Although, our performed measurements are fully equivalent to previous measurements by Fry *et al.* [5, 25, 26], it has to be pointed out that these are the first measurements with the proposed fiber amplifier and constitute an important milestone on the way to a practical implementation of the whole LIDAR system [14]. Despite stability limitations of our FPI, only 17.7 μJ of 532 nm radiation is sufficient to measure the Brillouin shift accurately.

## 4. Receiver

**Figure 6.**
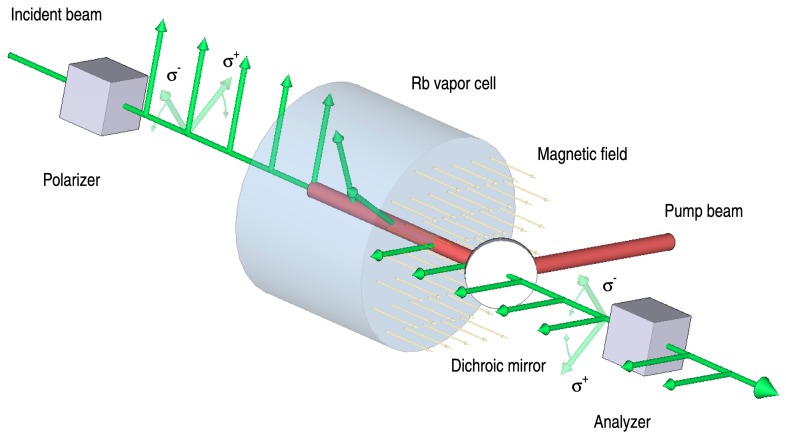
Schematic drawing of an ESFADOF consisting of an atomic vapor cell placed between two crossed polarizers. Due to the presence of the magnetic field, left and right circularly polarized light, σ^−^ and σ^+^, experience different dispersion in the vicinity of the atomic absorption lines. This results in a significant phase difference between the two polarization components. Therefore, a wavelength dependent rotation of the polarization occurs. The second polarizer projects the rotated polarization onto its basis rotated by 90° with respect to the first one. Therefore an ESFADOF delivers in addition a suppression of daylight.

The measurement of the Brillouin shifts will be based on a static edge-filter, capable of transforming these small shifts in frequency into large changes of the transmitted intensity. The design criteria of our edge filters can be summarized as follows: (1) Steep transmission edges, (2) symmetrically located in the spectral region of interest, i.e. ±7-8 GHz around the working wavelength, and (3) a maximum overall transmission with a sufficient signal-noise ratio. The symmetry significantly reduces the influence of laser frequency jitter. Several approaches are possible: Fabry-Perot etalons [16], molecular absorption lines [27, 28] or Excited State Faraday Anomalous Dispersion Optical Filter (ESFADOF). Although used in the present study, the Fabry-Perot is simply unsuitable for a practical implementation due to its very small angular acceptance. Molecular absorption lines have limited flexibility, especially when trying to use them as edge filters for both of the Brillouin shifted components simultaneously. The ESFADOF has a large angular acceptance provided by molecular absorption lines but also has the advantage of tunability for the positions of the filter edges. The ESFADOF is the approach that we are following. For completeness, we will briefly discuss the key ideas, for a more detailed characterization and discussion we refer to our earlier publications [10-12, 14, 15]. Briefly speaking Faraday anomalous dispersion optical filters (FADOFs) and their excited state counterparts ESFADOFs consist of an atomic vapor cell placed in a homogeneous magnetic field between two crossed polarizers. The working principle is as follows (cf. Figure 6): The incident light is polarized by the first polarizer and passes the gas cell. The high anomalous dispersion of the atomic vapor in the vicinity of its absorption lines results in a wavelength dependent rotation of the polarization vector. Finally, the second polarizer, which is rotated by 90°with respect to the first one, converts the rotation of the polarization into a change in the transmitted intensity. As ESFADOFs exploit atomic transitions between two excited states, an external pump source populating the lower ESFADOF state is necessary. Specifically, we investigated the 5P_3/2_ → 8D_5/2_ transition in Rubidium as the ESFADOF transition [12, 14]. Our results indicate that an edge filter based on this transition is possible. In order to push the edges to the required separation a magnetic field of B_Z_=500 mT is necessary. Currently, we are implementing a new cell design which will enable us to apply a field of this magnitude.

## 5. Conclusions and Outlook

We have presented for the first time data proving that accurate measurement of the water temperature by Brillouin scattering employing a frequency doubled fiber amplifier is possible. This is particularly challenging as the need for Fourier transform limited pulses reduces the threshold of stimulated Brillouin scattering in the fibers. Consequently, the pulse energy is limited to much lower values compared to those reported in the literature with much wider bandwidth pulses. Our temperature results agree well with previous measurements. The ESFADOF fulfills the basic requirements as a sensitive detection unit. The next steps will be depth resolved measurements as well as improvements to the ESFADOF, i.e. pushing the position of the edges to a separation suitable for actual Brillouin measurements.

## Figures and Tables

**Table 1.** Fiber properties of the three stages of the fiber amplifier. NA is the numerical aperture.

stage	1	2	3
core diameter	4.4 μm	28 μm	55 μm
inner cladding dia.	-	400 μm	400 μm
NA core	0.15	0.06	0.22
NA inner cladding	-	0.38	0.38
length	0.88 m	12.5 m	0.67 m
Yb concentration	6500 ppm	500 ppm	6500 ppm
